# Meta-Analysis of Pre-Clinical Studies of Early Decompression in Acute Spinal Cord Injury: A Battle of Time and Pressure

**DOI:** 10.1371/journal.pone.0072659

**Published:** 2013-08-23

**Authors:** Peter E. Batchelor, Taryn E. Wills, Peta Skeers, Camila R. Battistuzzo, Malcolm R. Macleod, David W. Howells, Emily S. Sena

**Affiliations:** 1 Florey Institute of Neuroscience and Mental Health, Heidelberg, Victoria, Australia; 2 Department of Medicine, University of Melbourne, Heidelberg, Victoria, Australia; 3 Division of Clinical Neurosciences, University of Edinburgh, Edinburgh, United Kingdom; University of Toronto, Canada

## Abstract

**Background:**

The use of early decompression in the management of acute spinal cord injury (SCI) remains contentious despite many pre-clinical studies demonstrating benefits and a small number of supportive clinical studies. Although the pre-clinical literature favours the concept of early decompression, translation is hindered by uncertainties regarding overall treatment efficacy and timing of decompression.

**Methods:**

We performed meta-analysis to examine the pre-clinical literature on acute decompression of the injured spinal cord. Three databases were utilised; PubMed, ISI Web of Science and Embase. Our inclusion criteria consisted of (i) the reporting of efficacy of decompression at various time intervals (ii) number of animals and (iii) the mean outcome and variance in each group. Random effects meta-analysis was used and the impact of study design characteristics assessed with meta-regression.

**Results:**

Overall, decompression improved behavioural outcome by 35.1% (95%CI 27.4-42.8; I^2^=94%, p<0.001). Measures to minimise bias were not routinely reported with blinding associated with a smaller but still significant benefit. Publication bias likely also contributed to an overestimation of efficacy. Meta-regression demonstrated a number of factors affecting outcome, notably compressive pressure and duration (adjusted r^2^=0.204, p<0.002), with increased pressure and longer durations of compression associated with smaller treatment effects. Plotting the compressive pressure against the duration of compression resulting in paraplegia in individual studies revealed a power law relationship; high compressive forces quickly resulted in paraplegia, while low compressive forces accompanying canal narrowing resulted in paresis over many hours.

**Conclusion:**

These data suggest early decompression improves neurobehavioural deficits in animal models of SCI. Although much of the literature had limited internal validity, benefit was maintained across high quality studies. The close relationship of compressive pressure to the rate of development of severe neurological injury suggests that pressure local to the site of injury might be a useful parameter determining the urgency of decompression.

## Introduction

Most human acute spinal cord injuries (SCI) are accompanied by significant on-going compression as a result of fractures, dislocations and associated trauma to the vertebral column [1,2]. A longstanding question has been whether prompt relief of this compression improves clinical outcomes in patients with SCI. Systematic reviews of the pre-clinical data have concluded that there is compelling evidence that early decompression improves outcomes in animal models of compressive SCI [3–9]. Recent human studies examining early decompression within 24hrs of injury have suggested a substantial benefit in around 15-20% of patients [10–13], while studies evaluating the effects of decompression beyond this time have been negative [14–19].

Despite this evidence, there is not yet consensus as to whether early decompression should be undertaken. While this is in part because the human data are not yet conclusive, it is also because of uncertainties in the interpretation of the pre-clinical literature. Pre-clinical studies use different methodologies and forming an overall estimate of the effectiveness of decompression is difficult, as is determining the extent to which the pre-clinical literature might be at risk of bias. An additional area of uncertainty is the timing of decompression, with some studies suggesting benefit only if compression is relieved within minutes, while others demonstrating decompression is effective even after many hours. Also unclear is what degree of canal compromise is required to cause significant compression and whether the window in which decompression is effective varies with this.

To address these questions we conducted a systematic review and meta-analysis of the pre-clinical literature on spinal cord decompression with particular emphasis on the relationship of outcome to the force and duration of compression. 

## Methods

### Systematic Review

In December 2011 electronic searches were performed on three separate databases; PubMed, ISI Web of Science and Embase. The following search strategy was employed to identify all possible publications: (decompression OR compression OR canal narrowing) AND (spinal cord injury OR contusion injury); search results were limited to animal studies. The review protocol entitled ‘Systematic review and meta-analysis of decompression in animal models of traumatic spinal cord injury’ can be found on the CAMARADES website at www.camarades.info/index_files/Protocols.html

### Inclusion and Exclusion Criteria

Studies for inclusion were screened by three independent reviewers (TW, ES and PB). To be included, studies must have reported the efficacy of decompression at different time intervals in an *in vivo* animal model of SCI. For inclusion in the systematic review, studies must have reported a behavioural outcome, lesion size or volume of preserved white matter. For inclusion in the meta-analysis, studies must have reported the number of animals, the mean outcome and the variance in each group. In each experiment we identified the control group to be the experimental group where compression was maintained for the longest period. Studies that did not describe such a group were excluded. For this reason, studies evaluating the effect of different compressive forces at a single time point were not included.

Studies examining decompression following injury using methods other than trauma (e.g. models of malignancy or disc herniation), and individual case reports describing outcomes from veterinary procedures for decompression were excluded. Meta-analysis was not conducted on histological or electrophysiological outcomes because these were performed too infrequently and variably for analysis to be reliable.

### Data collection

Reported behavioural outcomes, lesion size volumes and/or volume of preserved white matter were entered into the CAMARADES data manager (TW). In studies reporting more than one experiment, each experiment was considered as independent and data extracted for each, ensuring that correct weighting was provided in meta-analysis to reflect the number of experimental groups assessed for each control group. Where multiple behavioural outcomes were reported, data were extracted for each test. If numerical behavioural data were not available within the text, we extracted the data values and associated variance from the figures presented. Where the mean in the sham data was presented, this was taken to represent the outcome for uninjured animals. Where sham data were not available, pre-injury baseline data were extracted or inferred where possible (i.e. 21 on the BBB scale). Experimental animal species, sex and age were also extracted, as well as additional publication information including type of publication (abstract or full publication article), date and funding source. Data entries were checked by an independent investigator (PB) with any disagreements resolved via discussion with a third person (ES). Study quality was assessed according to the CAMARADES quality checklist, adapted from the consensus statement ‘Good Laboratory Practise’ in the modelling of stroke [20]. One point was given for each of the following items included in the checklist; (i) publication in a peer reviewed journal; (ii) statement describing control of temperature; (iii) randomisation to treatment group; (iv) allocation concealment; (v) blinded assessment of outcome; (vi) avoidance of anaesthetics with known marked intrinsic neuroprotective properties; (vii) sample size calculation; (viii) compliance with animal welfare regulations; (ix) and whether the authors declared any potential conflict of interest.

### Meta-analysis

For each experimental comparison reporting a behavioural outcome, a normalised effect size for decompression was calculated as the percentage improvement compared with outcome in the control (i.e. longest duration of compression) group. If the same group of animals were assessed using several different neurobehavioural scores in the one study, a summary estimate of efficacy in those animals was derived using fixed effects meta-analysis of the individual outcomes, and this summary was carried forward for further analysis. The DerSimonian and Laird weighted mean differences random effects model was used to aggregate the normalised effect size from each individual comparison.

Based on examination of the decompression literature and previous studies utilising meta-regression in SCI and stroke, we hypothesised that treatment specific parameters (compressive pressure and duration as well as the presence of co-treatment), model specific parameters (level of injury, method of compression, modelling paradigm, species, and anaesthetic agent), outcome specific parameters (neurobehavioural scale and time of final assessment) and measures to reduce experimental bias (blinded assessment of outcome, allocation concealment, randomisation, and sample size calculation) would influence outcome. The extent to which these study design characteristics explained differences between studies (study heterogeneity) was assessed using meta-regression with the *metareg* function of STATA/SE10 with the significance level set at p<0.05. Because the duration of compression used in experiments varied from seconds to 72 hours, meta-regression of compressive time against effect size was not valid. To adjust for the varying durations of compression and enable analysis by linear meta-regression, time points within each experiment were converted to percentages of the duration of the control group. The overall amount of heterogeneity is presented as an I^2^ value; 0-50% reflects low heterogeneity; 50-75% reflects moderate heterogeneity; and >75% reflects high heterogeneity. The extent that study characteristics account for between study heterogeneity is presented as the adjusted R^2^.

Evidence of publication bias was assessed using a funnel plot and Egger regression. We estimated the likely effect size in the absence of publication bias using the trim and fill method in STATA.

### Regression analysis

To determine the relationship between compressive pressure and the duration of compression that results in paraplegia, the pressure applied to the spinal cord in each experiment was estimated. Where studies reported the compressive pressure this was extracted from the methods section. In studies where the applied force was known (e.g. a 20g aneurysm clip), the compressive pressure was calculated from the area in contact with the spinal cord. In studies where a spacer or similar method was used to narrow the spinal canal the pressure was estimated by reference to the graphs of pressure versus canal diameter in Batchelor et al. (2011). Two different reference graphs were used depending on whether the cord had an initial contusion injury or not. The estimated compressive pressure was then plotted against the mean duration of compression necessary to produce severe neurological injury, defined as definite non-weight bearing locomotion. This outcome was chosen because non-weight bearing locomotion could be reasonably identified regardless of the neurobehavioural test used.

## Results

### Study Characteristics

**Figure 1:**
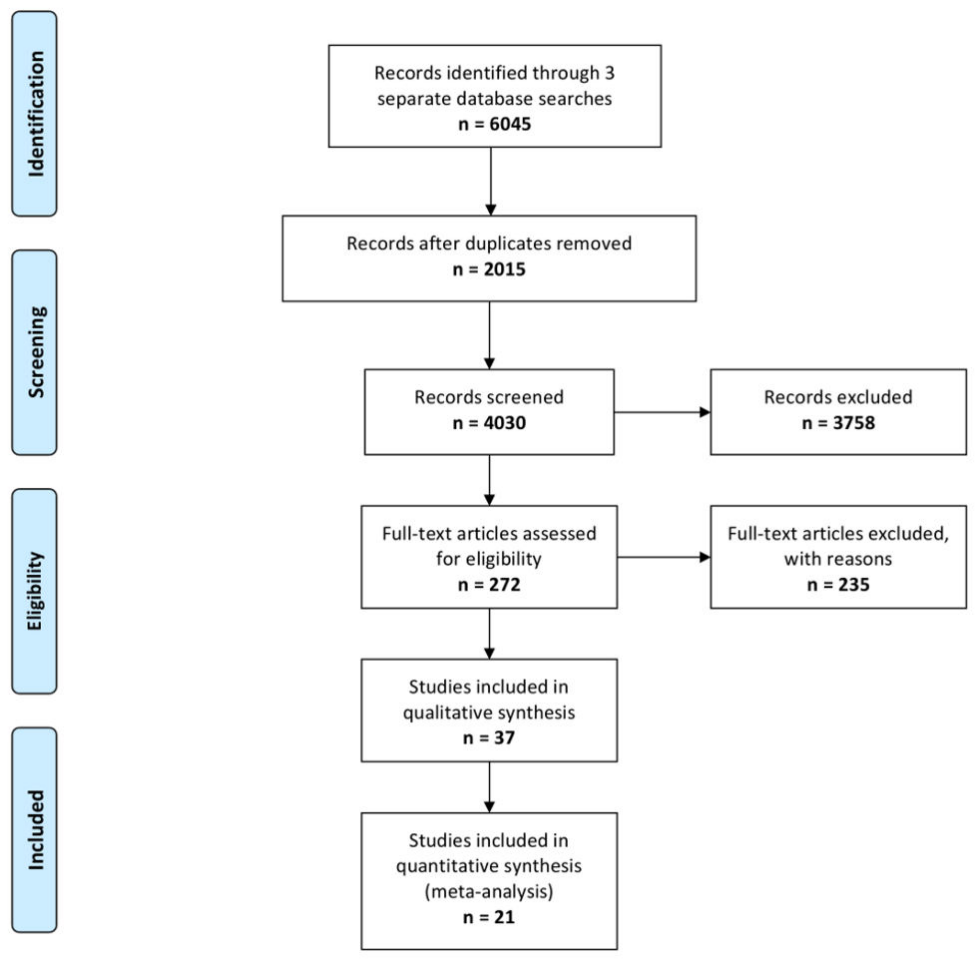
Flow diagram depicting the number of publications initially identified, number of records following removal of duplicates and exclusions, and the final number of publications included for analysis. Image adapted from: Moher D, Liberati A, Tetzlaff J, Altman DG, The PRISMA Group (2009). *P*referred *R*eporting *I*tems for *S*ystematic Reviews and *M*eta-*A*nalyses: The PRISMA Statement. PLoS Med 6(6): e1000097. doi: 10.1371/journal. pmed1000097.

Our systematic search identified 6045 publications. After removal of duplicate studies (n = 2015) and screening of titles and abstracts we retrieved 272 publications (Figure 1). Thirty-seven studies met the pre-specified inclusion criteria. Twenty one publications were suitable for meta-analysis [21–41]. The remaining 16 studies did not report sufficient data to be included in the meta-analysis and contributed only to the systematic review [32,42–56].

**Figure 2:**
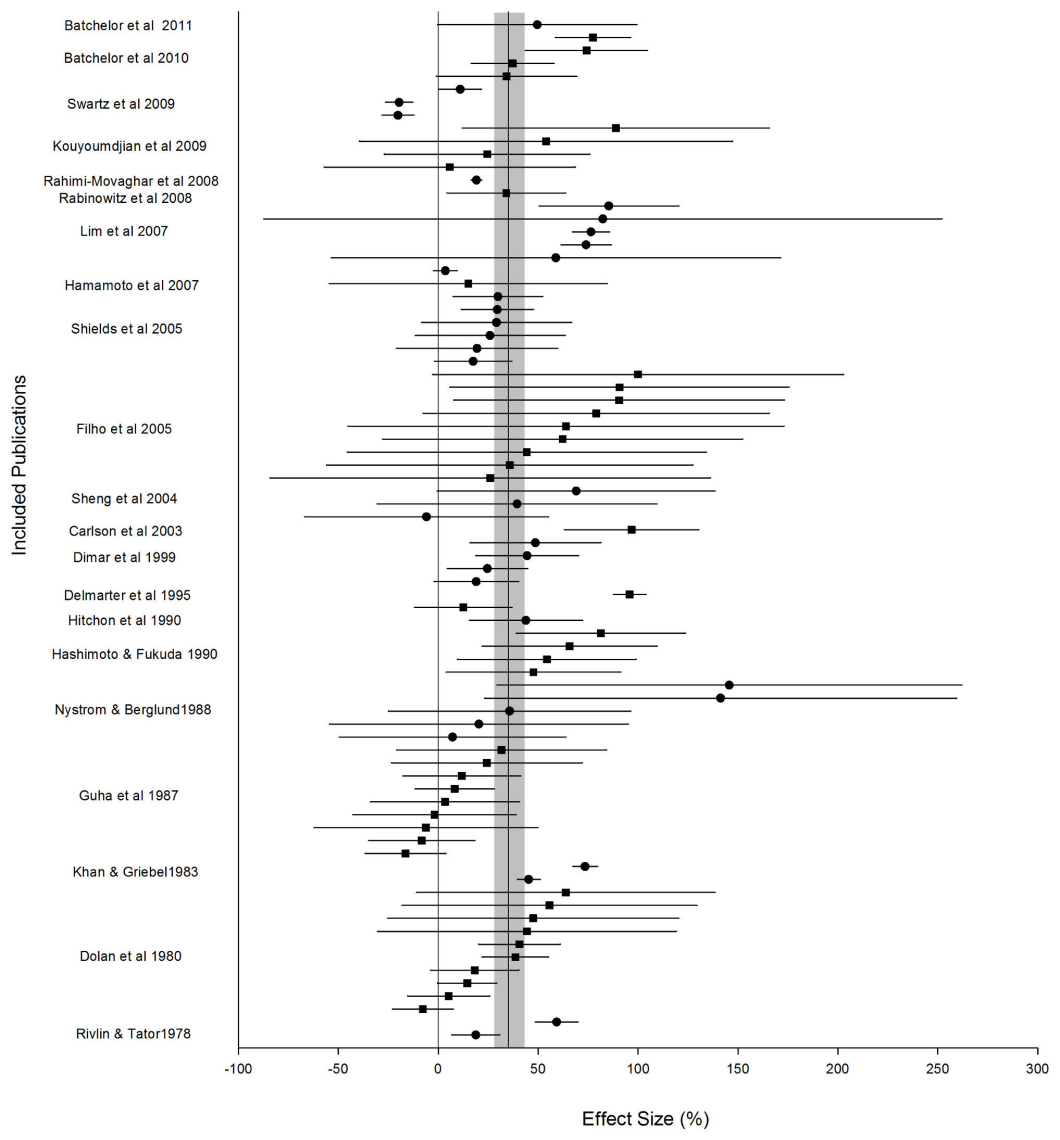
Effect size and 95% confidence intervals of the neurobehavioural assessments in the 79 experiments included in meta-analysis. The reference line represents the overall effect size of 35.1% with the gray shaded bar the 95% confidence intervals (27.5-42.8) of the global estimate.

Publications included in the meta-analysis contained a total of 79 separate experiments (using 873 animals) investigating the neurobehavioural effects of decompression after SCI, with several publications presenting multiple experiments. The overall effect size of the improvement in neurobehavioural outcome as a result of decompression was 35.1% [95%CI 27.4 to 42.8] and substantial heterogeneity was present (I^2^=94%, p<0.0001; Figure 2). Meta-regression was used to identify factors significantly influencing the effectiveness of spinal cord decompression.

### Treatment Specific Parameters

**Figure 3:**
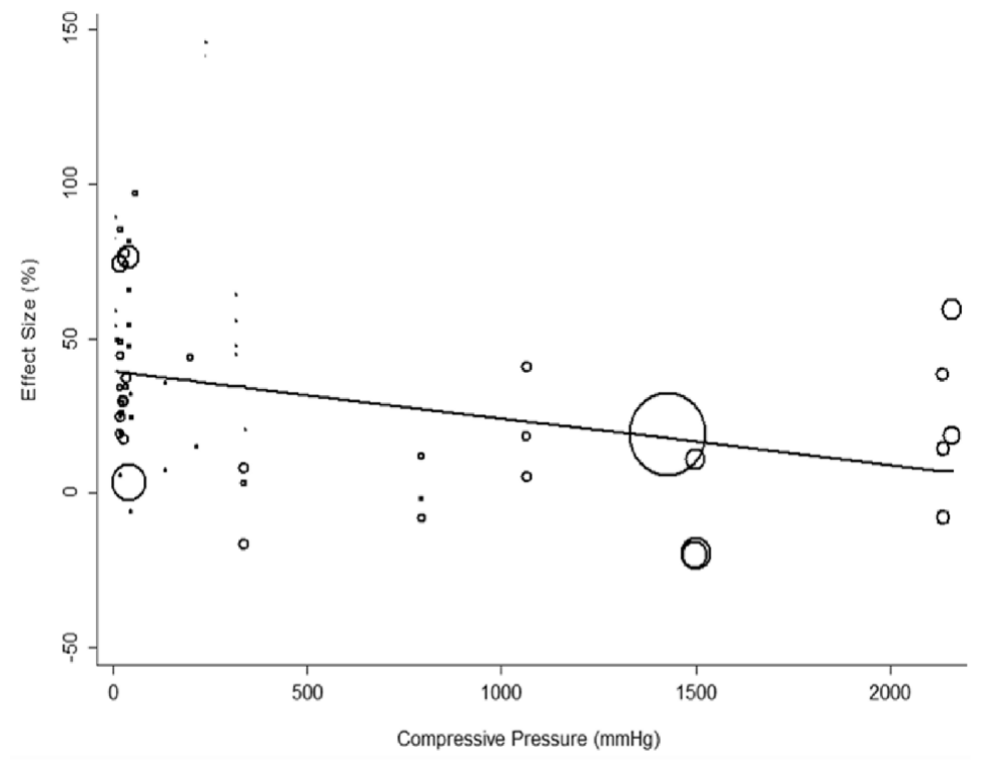
Meta-regression of functional (neurobehavioral) improvement versus compressive pressure (p=0.004). The size of each point reflects the precision of each comparison.

The degree of compression was difficult to compare directly across studies because of methodological differences. For example, some studies introduced spacers to narrow the canal diameter, others compressed the cord with aneurysm clips or weights exerting different forces, while a few studies compressed the cord with devices exerting known pressures. To compare the degree of compression between studies, the compressive pressure was calculated for each experiment. Compressive pressure (mmHg) was found to significantly influence neurobehavioural outcome, with higher pressures generally associated with a smaller effect size (p=0.004; Figure 3).

When examined by univariate analysis, the duration of compression was not significantly related to outcome (p=0.13). However, on multivariate analysis there was an inverse relationship between compressive pressure and the duration of compression (p=0.001). To explore the relationship between the force and duration of compression we recorded, for each cohort of animals, the period of compression necessary for those animals to develop clear paraplegia (defined as non-weight bearing locomotion), and compared this, in each case, with the compressive pressure used. These parameters could be extracted from 20 experiments in 16 of the 21 studies included in the meta-analysis, but only in two of the 16 excluded studies.

**Figure 4:**
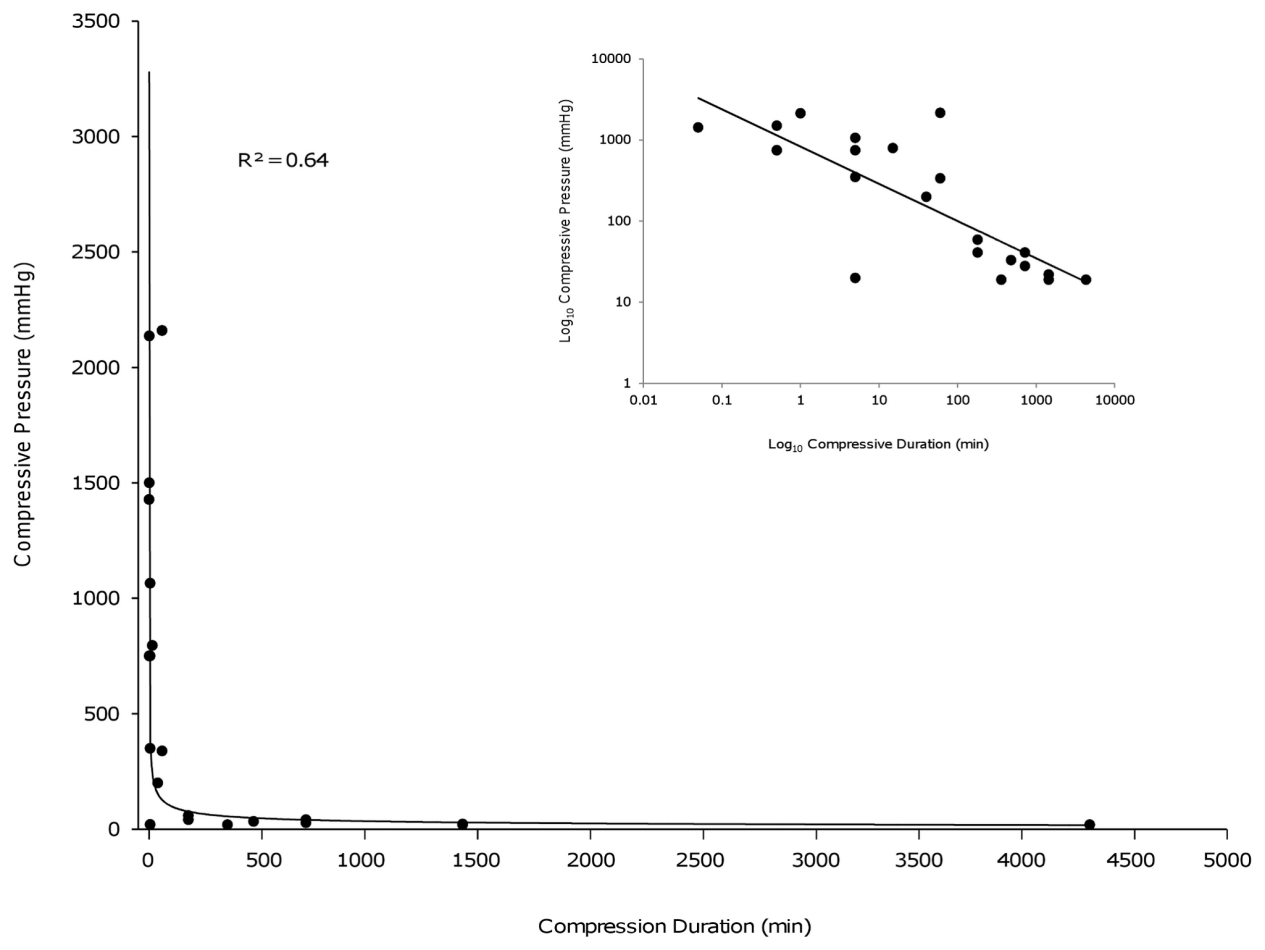
Line graph demonstrating the relationship between the duration of compression producing severe neurological injury and the compressive pressure in studies included in the meta-analysis. The association obeys a power law distribution (y = 743.17x^-0.443^), evidenced by a linear relationship on a log-log plot of the variables (inset).

Using these data, compressive pressure and compressive duration were associated by a power law (R^2^=0.64; Figure 4). A characteristic of this distribution is a linear association on a log-log plot of the variables (Figure 4 inset). This association suggests that as pressure increases the duration of compression necessary to produce severe neurological injury shortens increasing quickly.

**Figure 5:**
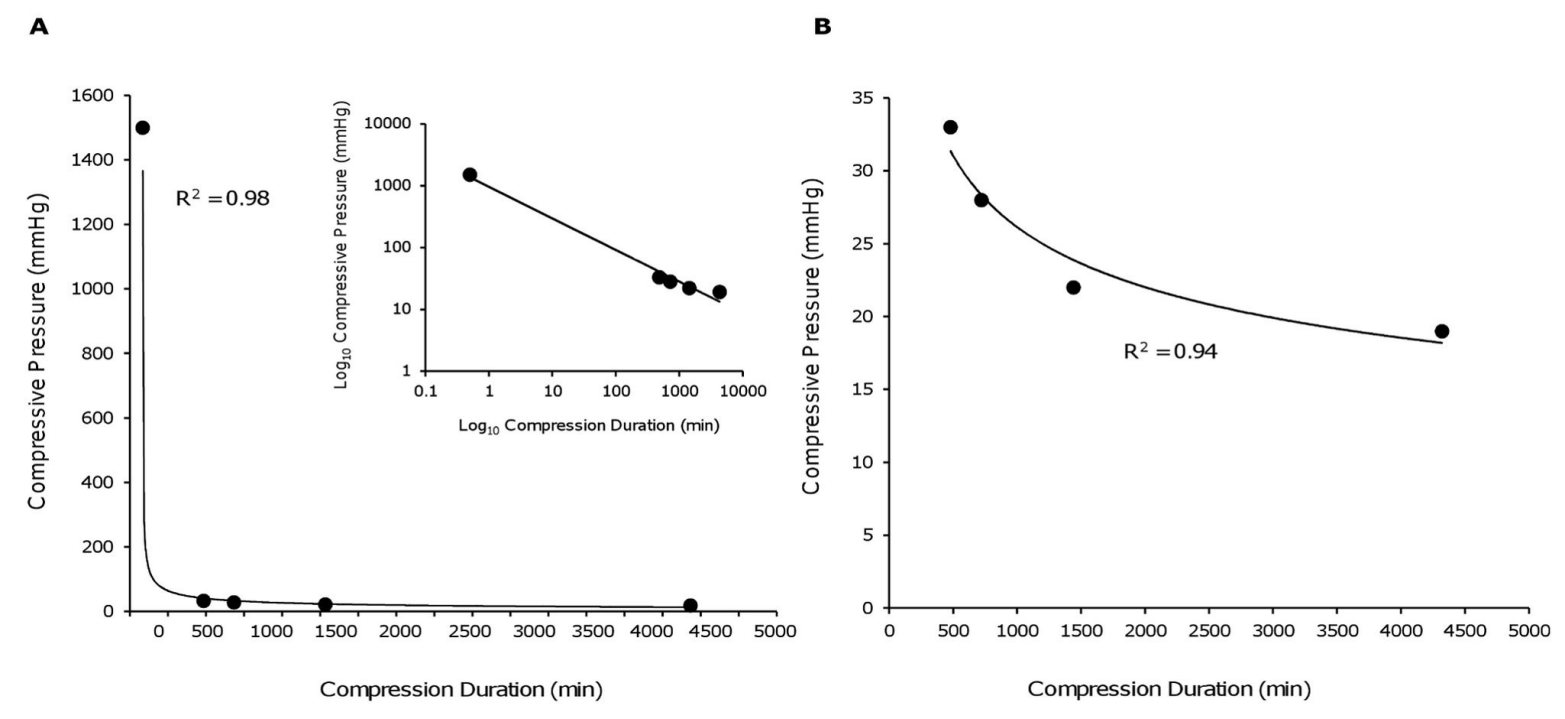
Line graph exploring the relationship between compressive duration and compressive pressure. (A) The association between the duration of compression producing severe neurological injury and the compressive pressure in those studies in which there was an initial injury to the spinal cord followed by compression. The data demonstrates a close correlation and again obeys a power law relationship (y = 829.06x^-0.459^) with a linear distribution on a log-log plot of the variables (upper inset). (B) Power law (y = 144.62x^-0.248^) relationship between compressive pressure and duration in studies employing an initial injury to the spinal cord followed by narrowing of the spinal canal to induce compression. These models had lower estimated pressures and longer durations of compression were necessary to produce paraplegia.

Although the data were closely correlated when all studies were included, the correlation between the data points was substantially greater when the analysis was limited to studies where an initial injury to the spinal cord was followed by compression (Figure 5A; R^2^=0.98). In models that utilised an initial injury to the spinal cord followed by the insertion of a spacer to narrow the spinal canal (which tend to replicate the human pattern of injury [25]), the curve of best fit again had a power law association, although at a different scale (Figure 5B; R^2^=0.93). The lower pressures generated in these models were associated with relatively long (8-72hrs) durations of compression before severe neurological injury was apparent.

The presence of a co-treatment (methylprednisolone or hypothermia) was not associated with a significant change in neurobehavioural outcome, although only 3 studies examined the effects of using a co-treatment with decompression [21,22,36].

### Model Specific Parameters

**Figure 6:**
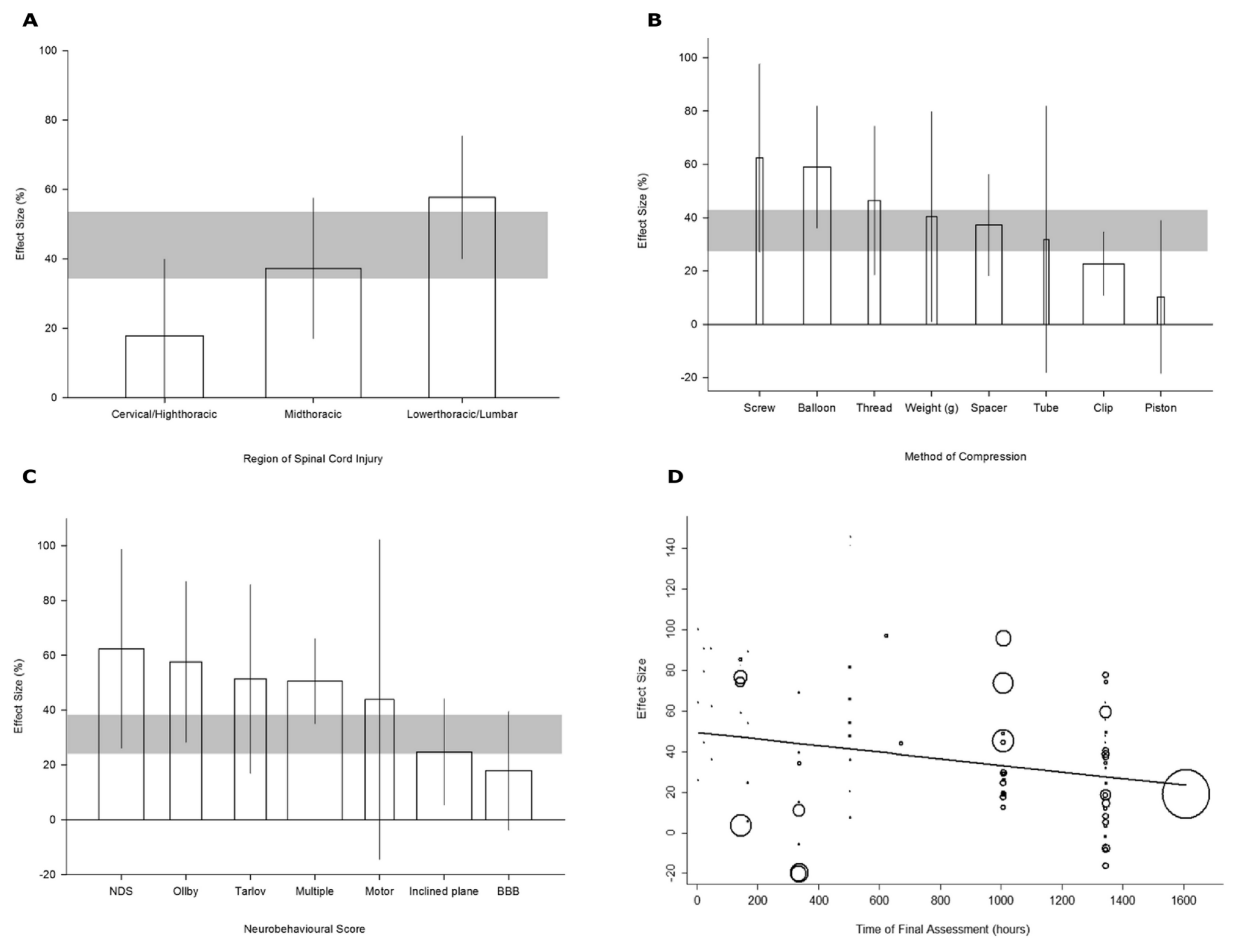
The change in effect size with (A) Region of injury, (B) Method of compression (Clip = aneurysm clip), (C) Neurobehavioural score (NDS = Neurologic deficit score; Olby = Olby score; Tarlov = Tarlov scale; Multiple = ≥2 behavioural tests; Motor = Motor test; BBB = Basso Beattie Bresnahan scale). The shaded gray bar represents the 95% confidence limits of the global estimate. The vertical error bars represent the 95% confidence intervals for the individual estimates. The width of each bar reflects the log of the number of animals contributing to that comparison. Each stratification accounts for a significant proportion of the heterogeneity observed between studies. (D) Meta-regression of functional neurobehavioural improvement versus the time of final assessment (p=0.046). The size of each point reflects the precision of each comparison.

We stratified the level of injury on the spinal cord into three groups (i) cervical and high thoracic injuries (C1-T4) (ii). mid-thoracic injuries (T5-T12) and (iii) lower thoracic and lumbar (T13 and below) injuries. Most experiments used mid-thoracic injuries. The benefit from decompression was inversely proportional to the level of injury (p=0.002; adjusted R^2^ = 17.5%; Figure 6A) with the greatest improvement in neurobehavioural outcome in lower thoracic/lumbar injuries (57.7% [40 to 75.4]), followed by mid-thoracic injuries (37.3% [17.1 to 57.5]) and then injuries to the cervical/high thoracic region (17.9% [-4.2 to 39.9]).

The method of compression significantly influenced neurobehavioural outcome (adjusted R^2^=17.2%; p=0.02). Compression with a screw or a balloon was associated with the largest neurobehavioural improvement (62.4% [27.2 to 97.7] and 59% [36.2 to 81.8%] respectively) with piston compression reporting only a 10.3% improvement [18.3 to 38.9]. Use of an aneurysm clip, balloon compression and spacer were the more common methods, with the least number of studies applying tube or piston compression (Figure 6B).

Four different animal species (dog, mouse, rat and sheep) were used in experiments, with rats most commonly utilised (n=700 animals; 61 experiments). Although there was a trend for greater behavioural improvements in dogs (59.2% [38.3 to 80.2]; n=66, 10 experiments) compared to rats (30.2% [21.9 to 38.5]) and mice (35.1% [1.7 to 68.6]), results did not achieve significance (p = 0.06).

Two different modelling paradigms were used; an initial spinal cord contusion injury with subsequent compression (5 studies) or compression of the spinal cord alone (16 studies). There was no significant difference in neurobehavioural outcome between these two groups (p=0.22).

A total of seven different anaesthetic agents were reported. However, the choice of anaesthetic did not impact on neurobehavioural outcome (p = 0.29).

### Outcome Parameters

Seven different scales of neurobehavioural assessment were reported. The use of multiple tests, the Basso, Beattie, Bresnahan (BBB) scale, and inclined plane test were the most frequently used assessment regimes. The smallest improvements in effect size occurred in studies employing the inclined plane test (24.8% [5.4 to 44.1]) and the BBB scale (17.9% [-3.7 to 39.4]), while the use of multiple tests, the neurologic deficit score, Olby score and Tarlov scale were associated with the highest magnitudes of improvement (Figure 6C).

The time of final assessment of experimental animals ranged from a few days to months after the initial injury. The effect of decompression appeared to decrease as the time from the injury to final assessment increased (adjusted R^2^=1.1%) (p=0.046; Figure 6D).

### Experimental and Publication Bias

**Figure 7:**
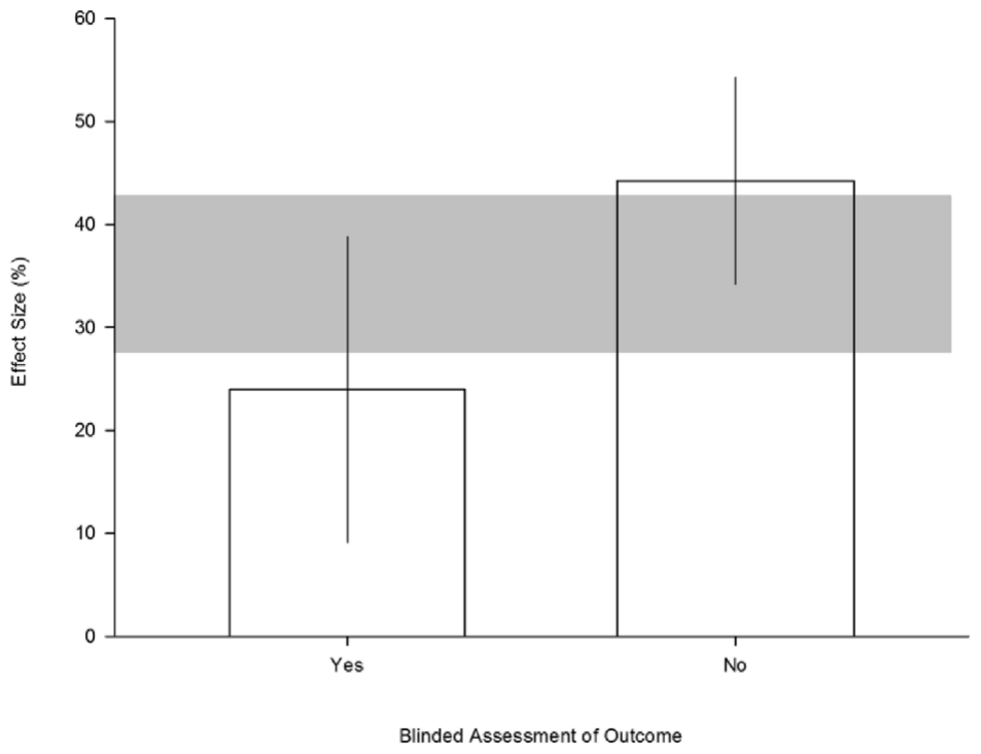
Effect of reported study blinding on effect size. The shaded gray bar represents the 95% confidence limits of the global estimate. The vertical error bars represent the 95% confidence intervals for the individual estimates. The width of each bar reflects the log of the number of animals contributing to that comparison.

We sought to determine the influence of measures to reduce experimental bias on neurobehavioural outcome. Less than half of the publications (9/21) reported blinded assessment of outcome. These blinded studies reported 20% smaller effect sizes than non-blinded studies (adjusted R^2^=10.2%) (24% [9.1-38.8] versus 44.2% [34.2 to 54.3]; p<0.008, Figure 7).

Only 3 publications reported allocation concealment. Seven of 21 studies (33.3%) reported random allocation to treatment group and 2 publications reported sample size calculations. Eight studies contained a statement of potential conflict of interest. These factors did not affect neurobehavioural outcome, although the small number of studies reporting these factors precludes confidence in the statistical analyses.

**Figure 8:**
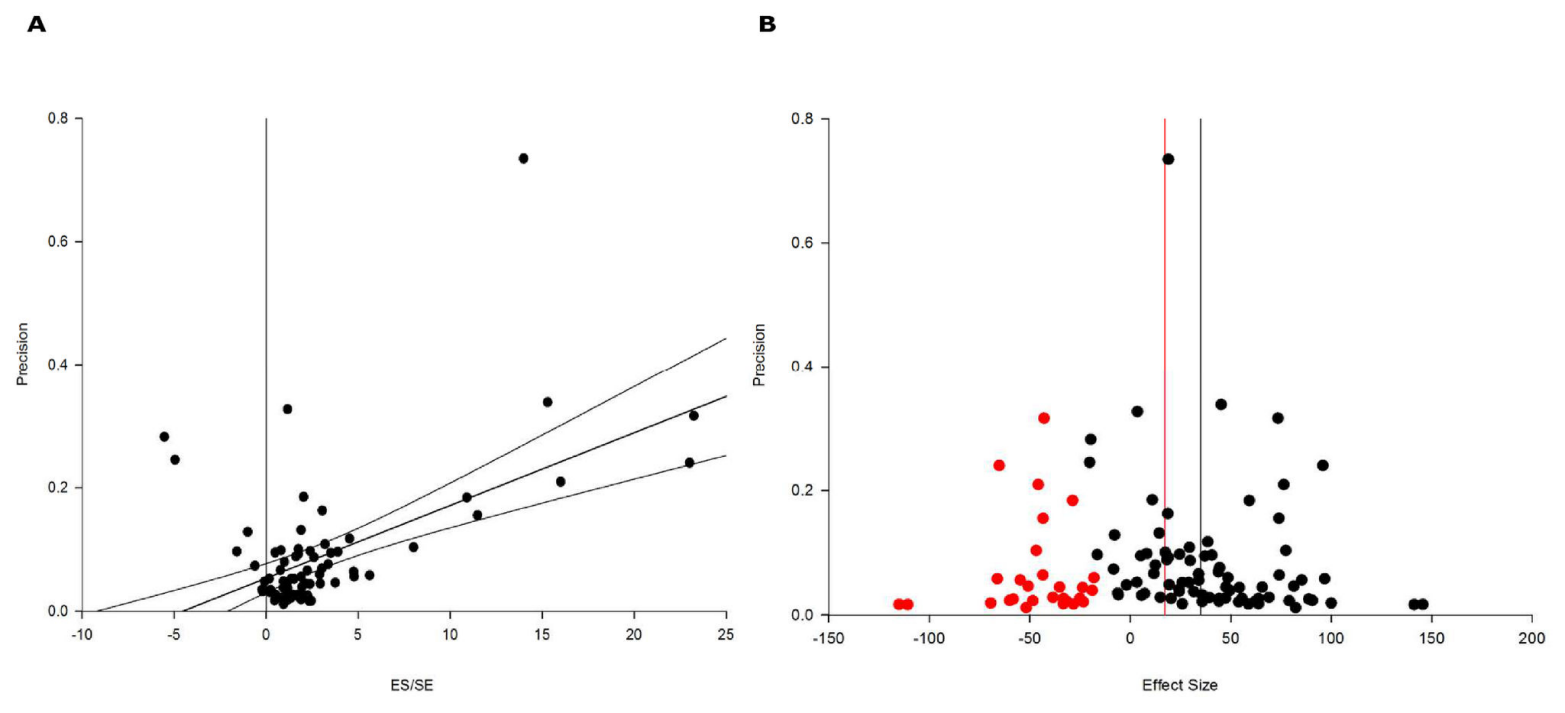
Evidence of publication bias demonstrated by (A) Egger regression analysis of early decompression experiments. The 95% confidence intervals of the regression line do not include the origin, suggesting the presence of a significant publication bias. (B) Funnel plot showing the data in black and the additional missing studies suggested by trim and fill in red. The red vertical line indicates the possible global estimate in the absence of publication bias.

Significant publication bias was apparent using Egger regression, with the 95% confidence intervals of the regression line not including the origin (Figure 8A). Trim and fill analysis also suggested the presence of publication bias and the possible absence of 29 negative experiments (Figure 8B) leading to an overstatement of efficacy of 18.5%.

### Studies Excluded from Meta-analysis

Sixteen of the 37 studies did not report sufficient quantifiable data to be included in the meta-analysis, although 2 studies [32,55] were able to be included in the regression analysis of compressive pressure versus duration. The quality of excluded studies (median quality score of 2) was lower than those included in the meta-analysis (median quality score of 4). These studies were undertaken in 5 different species (mouse, rat, rabbit, dog and primate) and all but one study reported positive effects of early decompression on neurobehavioural outcomes or the degree of tissue preservation. 

## Discussion

This study assesses the pre-clinical literature reporting acute decompression of the injured spinal cord using meta-analysis. The overall behavioural improvement following decompression was 35.1%, with all but one study included in the meta-analysis reporting a beneficial impact of decompression on behaviour. Sufficient heterogeneity was present between studies to allow the impact of individual factors on outcome to be evaluated using meta-regression. A number of factors emerged from this analysis as having an impact on outcome, including both the pressure and duration of compression.

### Relationship between Compressive Pressure and Duration

In univariate analyses the effect size and compressive pressure followed an inverse relationship, with higher pressures associated with smaller effects. The duration of compression was not related to outcome. However, in multivariate analysis of both the pressure and duration of compression we observed a strong relationship with outcome. Therefore, it appears that the duration of compression is an important factor in determining outcome, but only in relation to the compressive pressure; when the influence of pressure is removed, time ceases to be an important factor.

While meta-regression demonstrated an association between pressure and time, we sought to determine the nature of that relationship by comparing the force of compression in each study with the duration of compression necessary for animals to develop paraplegia. Interestingly, the association evident between these variables obeyed a power function. This association suggests that with low compressive forces (e.g. exerted by spacers narrowing the spinal canal) longer durations of compression are necessary to significantly affect outcome. In contrast, with high compressive forces (such as exerted by most aneurysm clips), only short durations of compression are necessary to produce the same severity of neurological injury. Considering the independent nature of the studies, the data demonstrate remarkable concordance. The strongest correlation was evident in data derived from experiments where there was an initial injury to the spinal cord followed by compression. Models that arguably most closely simulate the mechanism and timing of injury in humans are those employing fixed degrees of canal narrowing following an initial spinal cord contusion injury [21,25,40]. These models were associated with relatively low pressures and relatively long times to severe neurological injury.

These data suggest that the duration of compression resulting in poor outcome is critically dependent on the pressure applied to the spinal cord. The compressive pressures accompanying human injuries are unknown. However, experimental canal narrowing of a similar degree to that present in complete human injuries is accompanied by compressive pressures of around 30-35mmHg [22]. Pressure and canal narrowing of this magnitude applied to the injured rodent spinal cord results in significant deficits in 2-6 hours and severe paraparesis in 8-12 hours [21,25,40]. Ischaemia is an important mechanism of injury following compressive SCI [29,57–63] and equivalent ischaemic times in humans are around 2-3 times longer than those in rodents [64–67]. Together, this information suggests that decompression before approximately 12 hours post-injury in humans might result in substantial benefits, with a lesser degree of benefit occurring with decompression between 12 and 24 hours.

Although the degree of canal compromise is an important variable, in human injuries pressure is also likely to depend on other factors such as congenital canal diameter, cord oedema and haematomyelia [68–72]. Given that the relationship between force and time obeys a power law, small increases in pressure would be expected to rapidly increase the urgency of decompression. Conversely small reductions in pressure with therapies such as hypothermia [22] might lengthen the time available for decompressive surgery.

Because the compressive force applied to the injured spinal cord appears to dictate the rate of progression to severe neurological injury, measuring intracanal pressure local to the site of injury could potentially be clinically useful and allow patients to be better triaged according to the urgency of surgery. Non-invasive methods of determining pressure would be preferable and one potential approach might be to adapt MRI methods of measuring intracranial pressure [73,74].

### Modelling Compressive SCI

The data demonstrate that experimental models using very high compressive forces generally result in paraplegia with durations of compression measured in minutes. In contrast, the duration of compression needed to produce paraplegia in animal models with relatively low compressive forces (such as canal narrowing) is many hours (Figure 3). This latter timeframe seems to concord better with the human timeframe of injury, with clinical data suggesting that early decompression is of benefit in at least a proportion of patients when performed 6-20 hours post-injury [10–12]. These data support the use of low compressive pressures when modelling the effects of early decompression on acute SCI. However, power curves have the useful property of allowing accurate extrapolation [75]; thus times relevant to human injury might be obtained if data is fitted to a power curve and extrapolated to the range of lower pressures likely to accompany human injury.

Of the species employed in pre-clinical studies of early decompression, a trend towards a larger effect size was seen in studies using dogs. However, this may simply reflect the different neurobehavioural scales used in each species as well as differing study designs. For example, the duration of compression tended to be shorter in dog studies, while an initial injury prior to compression of the spinal cord was present in a number of rodent studies. Supporting this interpretation, when these effects were removed and just the time to paraplegia examined in relation to the duration of compression, the data regardless of whether derived from a mouse, rat, dog, sheep or primate model fell around the same curve (Figure 2). The consistent relationship of the data regardless of species suggests that small animal models are equally as valid as their larger counterparts, at least for modelling compressive SCI.

Compressive injury to the cervical/high thoracic region was accompanied by the smallest effect size. This may reflect the use of the inclined plane test to assess neurobehavioural recovery in these studies, without separate assessments of forelimb recovery. Relatively poorer performance on the inclined plane test would be expected with both forelimb and hindlimb function affected compared to the majority of other studies where impairments were confined to the hindlimbs.

Outcome appeared to be significantly affected by the method of compression. However, although piston compression had a smaller overall effect size there were only two studies utilising this method, one of which was positive, while the other was one of the few studies with negative findings. This is reflected in the wide confidence interval.

A generally smaller effect size was observed as the interval to final assessment increased. This may in part reflect the slower and more protracted pattern of recovery by animals with severe injuries. In some studies, differences between animals with the most severe injuries and those animals with milder injuries as a result of earlier decompression were relatively greater initially, before decreasing as the more severely injured animals recovered [21–25,28]. Methodological differences may also be responsible; those studies with long assessment times more often had injuries in the cervical/high thoracic region, an initial injury to the spinal cord prior to commencement of compression, or evaluated behaviour using the BBB and inclined plane tests, all factors associated with at least trends to smaller effect sizes.

Only two of the included studies found no benefit from decompression. In the meta-analysis group, 1/21 studies were negative, while in the group excluded from meta-analysis 1/16 reported negative findings. Variations in the animal models may account for the findings in these studies. Lee et al. (2008) employed a balloon occlusion model, with the balloon completely occluding the spinal canal for 30 or 60 minutes. This likely resulted in a very high compressive pressure and no recovery of the animals was reported. Swartz et al., (2009) employed a model with an initial injury followed by a very high compressive force for varying short periods (10s-5min). Although decompression was found to be of benefit overall, no difference was reported between the groups with different durations of compression. The reasons for this result are unclear and different explanations have been proposed [76]. The traumatised cord is particularly vulnerable to compression [25] and it may be that even a very short duration of high pressure compressive injury creates a maximal lesion.

### Study Quality and Publication Bias

A number of investigations have demonstrated the importance of study quality, with a decrease in effect size consistently observed as quality improves [20,77–80]. Although 7 decompression studies had quality scores of 5 or more [21,22,29,33,37,40,41], the overall quality of the dataset was modest, with a median quality score of 4 in studies included in the meta-analysis and 2 for those studies excluded. Blinding is a key factor in maintaining the internal validity of an experiment but was only reported in approximately half of decompression studies. Those studies reporting blinding were associated with a significantly smaller effect size (24% vs. 44%). Relatively few studies reported other key factors required to minimise the introduction of experimental bias including randomisation, concealed allocation and sample size calculations. Statistical analysis was impaired by the small number of studies reporting these items.

An important finding was the potential presence of publication bias. The funnel plot of the data was not symmetrical, with trim and fill analysis showing the possible absence of 29 negative experiments from the published literature. However, these data do not definitively prove the presence of publication bias and an alternative interpretation is simply that low precision experiments are sometimes associated with exaggerated estimates of efficacy. Regardless of the explanation, these results are in keeping with an overstatement of efficacy in the early decompression literature, an interpretation supported by the reduction in efficacy seen as study quality improves.

### Study Limitations

This meta-analysis is weakened by the overall modest study quality and the possibility that a considerable amount of data remain unpublished. Although the search strategy is likely to have ascertained the majority of relevant publications it is possible that some studies were not retrieved. We would have liked also to examine histological outcomes using meta-analysis. However, the limited number of studies reporting quantitative histology prevented this. The approach is correlative and observational rather than experimental and therefore limits the ability to draw definite conclusions.

## Conclusion

Meta-analysis of the pre-clinical literature suggests that early decompression is an effective therapeutic strategy. The majority of studies report positive findings, with an overall estimated effect size of behavioural improvement following decompression of 35.1%. The true effect size may, however, be smaller than this, as blinded assessment was associated with a significant reduction in effect size (24%) and publication bias appears to be present. Outcome following acute compressive spinal cord injury appears to be closely tied to the compressive pressure and duration. As compressive pressure rises, the duration of compression necessary to produce severe neurological injury rapidly shortens. The close relationship of compressive pressure to the rate of development of severe paraplegia suggests that pressure local to the site of injury may be a useful, potentially measurable parameter to determine the urgency of decompressive surgery.

## Supporting Information

Checklist S1 **Prisma checklist.**
(DOC)
